# First report of the presence of L1014S Knockdown-resistance mutation in *Anopheles gambiae s.s *and *Anopheles coluzzii* from Togo, West Africa

**DOI:** 10.12688/wellcomeopenres.13888.1

**Published:** 2018-03-19

**Authors:** Innocent Djègbè, Romaric Akoton, Genevieve Tchigossou, Koffi Mensah Ahadji-Dabla, Seun Michael Atoyebi, Razack Adéoti, Francis Zeukeng, Guillaume Koffivi Ketoh, Rousseau Djouaka

**Affiliations:** 1 University of Sciences, Technologies and Mathematics, Ecole Normale Supérieure de Natitingou, Natitingou, BP 123, Benin; 2 The AgroEcoHealth Platform, International Institute of Tropical Agriculture, Cotonou, 08 BP 0932, Benin; 3 Faculty of Sciences and Techniques, University of Abomey-Calavi, Cotonou, BP 526, Benin; 4 Research unit of Ecotoxicology, Faculty of Sciences, University of Lomé, Lomé, 01 BP 1515 , Togo; 5 Department of Biochemistry, Faculty of Sciences, University of Yaoundé I, Yaoundé, BP 812, Cameroon

**Keywords:** Insecticides resistance, Anopheles gambiae s.s., Anopheles coluzzii, kdr mutation, Ace.1R mutation, Togo.

## Abstract

**Background: **To optimize the success of insecticide-based malaria control intervention, knowledge of the distribution of *Anopheles gambiae *species and insecticide resistance mechanisms is necessary. This paper reported an updated data on pyrethroids/DDT resistance in the *An. gambiae s.l* population from Togo.

**Methods: **From December 2013 to April 2015, females of indoor-resting *An. gambiae s.l* were captured in three locations belonging to three different ecological zones. Resistance to DDT, permethrin and deltamethrin was screened in F1 progeny of collected mosquitoes using WHO susceptibility tests. The identification of species of *An. gambiae* complex and the detection of *kdr* and *ace.1 ^R^* allele were carried out using DNA-based molecular techniques.

**Results:** *An. gambiae* from Kovié and Nangbéto were highly resistant to DDT and permethrin with mortalities rate ranging from 0.83% to 1.58% for DDT and zero to 8.54% for permethrin. Mosquitoes collected in Nangbéto displayed 81.53% mortality with deltamethrin. *An. coluzzii* and *An. gambiae* s.s were found in sympatry in Nangbéto and Mango *. *The allelic frequency of L1014F was high, ranging from 66 to 100% in both *An. coluzzii* and *An. gambiae s.s*. For the first time we detected the L1014S allele in both *An. coluzzii *and *An. gambiae* *s.s.* from Togo at the frequency ranging from 5% to 13% in all the sites. The *kdr* N1575Y was present at various frequencies in both species ranging from 10% to 45%. Both *An. gambiae s.s.* and *An. coluzzii* shared the *ace1 ^R^* mutation in all investigated sites with allelic frequency ranging from 4% to 16%.

**Conclusion: **These results showed that multiple mutations are involved in insecticides resistance in *An. gambiae *populations from Togo including the kdr L1014F, L1014S, and N1575Y and *ace.1 ^R^* G119S mutations.

## Introduction

Despite a reported decline in infection and mortality, malaria remains the fourth leading cause mortality in children under-five in the sub-region ^1^. In Togo, the malaria control strategy is based on universal access to Long Lasting Insecticidal Nets (LLINs) as recommended by the World Health Organization (WHO). However, malaria vectors were found resistant to all insecticide classes used in public health interventions in West Africa. Mosquito resistance to insecticides stands as a serious obstacle to the effectiveness of LLINs. Since 2010, reports of pyrethroids and dichlorodiphenyltrichloroethane (DDT) resistance have been widespread ^2– 6^ with further reports of carbamate resistance ^7– 9^. The resistance to pyrethroids/DDT is conferred by two main physiological mechanisms including metabolic resistance and the target site insensitivity ^10^. The target site resistance remains the most studied to date, a result of the easier means of its assessment ^11^. In *Anopheles gambiae* complex, *kdr* gene mutations, including the substitutions of leucine to phenylalanine (L1014F) and leucine to serine (L1014S), are the two mutations involved in the target site resistance ^12, 13^. These two target site mutations are largely distributed across the African Continent, yet differences in the allelic frequency have been reported between *An. gambiae s.l.* species and between breeding sites ^3, 14, 15^. In several studies, clear associations has been shown between DDT/pyrethroids resistance and the presence of *kdr* mutations ^16^. Recently, a new mutation named N1575Y has emerged within the linker between domains III-IV of the voltage gate sodium channel (VGSC) in *An. gambiae s.s., An coluzzii* and *An. arabiensis* ^17^. Studies have demonstrated the appearance of the N1575Y mutation as an additional resistance mechanism that appears with the L1014F *kdr* mutation. N1575Y mutation is therefore being suggested as a secondary selective sweep, associated with resistance to pyrethroids/DDT in the West African region ^17, 18^. The development of new resistance mechanisms among *Anopheles* populations highlights a failure in pyrethroids-based control strategies, and could jeopardize the mosquito control efforts ^19^. It is therefore seminal to establish the epidemiological consequences of pyrethroids resistance, and develop new intervention strategies for the management of insecticides resistance in West Africa.

Monitoring the molecular and physiological markers of pyrethroids resistance has significant advantages for the management of insecticides resistance. In Togo, despite the huge investments in LLINs, information is still lacking on the routine monitoring of insecticides resistance in malaria vectors. An entomological survey was carried out since 2009 in two localities of the Southern coastal region (Lomé and Kovié) and revealed the involvement of *kdr* L1014F in insecticides resistance ^20^. Additional knowledge on the mechanisms involved in insecticides resistance is needed across the country, as this is seminal to design new strategies for insecticides resistance management. We therefore attempted in this study to investigate the distribution of the *kdr* L1014F, L1014S and N1575Y alleles and *ace.1 ^R^* mutation in three localities across the country using WHO bioassays and advanced molecular methods.

## Methods

### Study sites

**Figure 1.**
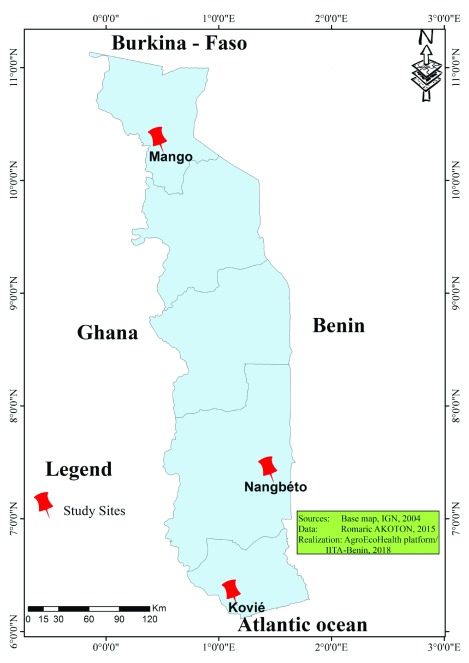
Map of Togo showing the study sites.

The study was carried out in three rural sites from different ecological zones of Togo ( Figure 1, Table 1). Togo is a coastal country located in West Africa, with a population of ~7 million inhabitants. The country has two tropical climates: the Sudanian in the North, characterized by one rainy season and one dry season; and the subequatorial in the South, characterized by two dry seasons (from December to March and from August to September) and two rainy seasons (from April to July and from October to November). According to Ahadji-Dabla *et al.* ^21^, the Republic of Togo covers five ecological zones: the plains zone in the North (zone I), the mountains zone in the North (zone II), the Central plains zone (zone III), the Mounts Togo meridional zone (zone IV), and the Southern coastal zone (zone V). Table 1 describes the characteristics and geographic coordinates (GPS) of the investigated sites.

**Table 1.** Characteristics of study sites.

Localities	Main agriculture practices	Ecological zones	Periods of collection	Geographic coordinates	
Kovié	Rice, vegetables	(Coastal)	December 2013	N 06°20.305'	001°07.425' E
Nangbéto	Cereals, tubers	(Forest)	April 2015	N 07°25.802'	001°26.822' E
Mango	Cereals, cotton	(Savannah)	April 2015	N 10°21’17.9’’	0°28’21.7’’E

### Mosquito collection and rearing

Mosquito sampling was conducted from December 2013 to April 2015 using electric aspirators. In each study sites, the households were randomly selected for mosquito aspirations. Verbal and written consents of the household heads were sought prior to insect collection in their houses. Indoor resting blood fed adult female *Anopheles* mosquitoes (F0), were captured between 06.00 and 10.00 am, kept in cool boxes and brought to the insectary of the AgroEcoHealth Platform of the International Institute of Tropical Agriculture (IITA-Benin). A forced-egg laying method was used to induce the females to lay eggs as previously described ^22^. The egg batches were then allowed to hatch in a small paper cup and later transferred to larvae bowls for rearing as previously described ^22, 23^.

### Insecticide susceptibility test

F1 female progeny of wild *An. gambiae* and laboratory susceptible Kisumu strain aged 3–5 days were exposed to impregnated papers at diagnostic concentrations of insecticides according to WHO protocol ^24^. Insecticide papers were obtained from the WHO reference centre at the Vector Control Research Unit, University Sains Malaysia. The impregnated papers included 4% DDT, 0.75% permethrin and 0.05% deltamethrin. Briefly, for each tested insecticide, batches of 20–25 unfed females were exposed to an impregnated paper for 60 min, after which they were transferred into tubes containing untreated papers and placed under observation at 25°C and 80% relative humidity (RH) with 10% sugar solution. Mortality rate was recorded 24h post-exposure. Tube tests containing untreated papers were run in parallel as a control.

### Species identification, kdr L1014F, L1014S, N1575Y and *ace.1 ^R^* genotyping

Genomic DNA from respectively 88, 70 and 58 females (F0) collected in Kovié, Nangbéto and Mango of bioassay control was individually extracted using the Livak DNA extraction method ^25^. The species of *An. gambiae* mosquitoes were identified using polymerase chain reaction techniques. The *kdr* mutations *L1014F*, *L1014S and N1575Y* were screened ** using TaqMan real time PCR assays as previously described ^17, 26^. The presence of G119S-Ace1 allele was also screened in *An. gambiae* populations as describe by Bass *et al*. ^27^.

### Statistical analysis

The resistance profile of *An. gambiae s.l.* was determined using WHO criteria ^28^:

Mortality rate > 98% = susceptible mosquito population

Mortality rate between 90–98% = suspected resistance in the mosquito population

Mortality rate < 90% = resistant mosquito population

According to Hardy-Weinberg equilibrium using the Had2know online statistical software, calculated genotype frequencies of L1014F, L1014S, N1575Y and G119S were confirmed and compared between *An. coluzzii* and *An. gambiae s.s.* with Chi-square test.

## Results

### Species composition of *An. gambiae s.l.*

**Figure 2.**
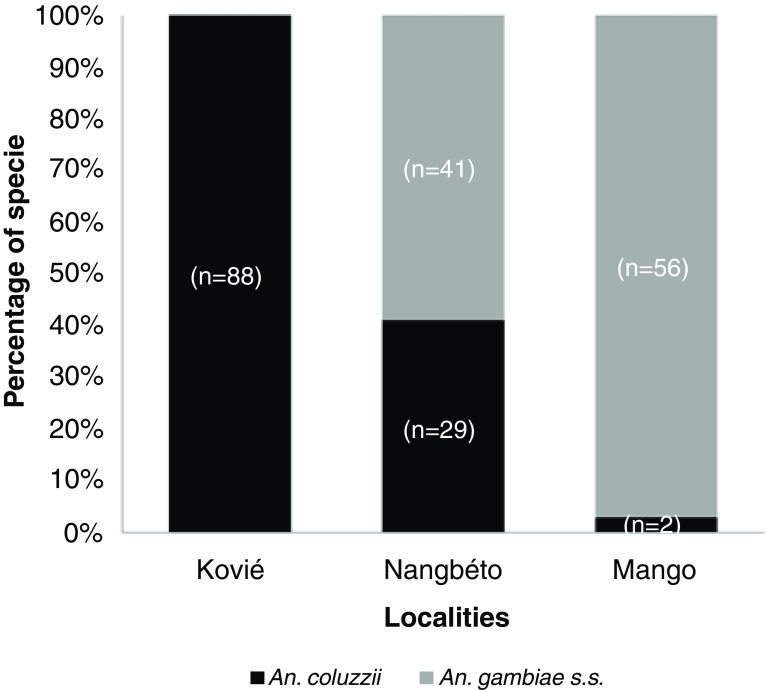
Species composition in study sites.

The PCR analysis on the F0 females for identification of sibling species among *An. gambiae* complex revealed the presence of only two species in the study sites as *An. coluzzii* and *An. gambiae s.s.*. Both *An. gambiae s.s.* and *An. coluzzii* were found in sympatry in Nangbéto (59% versus 41%) and Mango (97% versus 3%). *An. coluzzii* was predominant (100%) in Kovié (rice field) ( Figure 2).

### Insecticide resistance status

**Figure 3.**
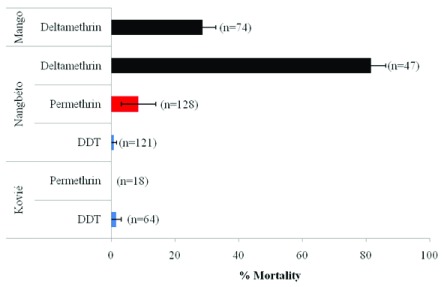
Insecticide resistance profiles of *An. gambiae s.l*. in Kovié, Nangbéto and Mango (Error bars are standard error).

Figure 3 describes the insecticide resistance profile of three *An. gambiae s.l* populations collected in Togo. The laboratory strain Kisumu exhibited very high susceptibility to the insecticides tested: 99% mortality to 100% mortality to the organochlorine and permethrin respectively, and 99.5% mortality to deltamethrin. In control groups (untreated papers) the mortality rates recorded with the wild *An. gambiae* populations were below 5% at 24 hours post-exposure.

*An. gambiae* population from Kovié and Nangbéto were highly resistant to DDT and permethrin with mortality rates ranging from 0.83% to 1.58% for DDT and from zero to 8.54% for permethrin. Mosquitoes collected in Mango and Nangbéto displayed high resistance to deltamethrin with mortality rates of 28.67% and 81.53% for permethrin and deltamethrin respectively.

### Detection of resistance genes

Mosquitoes from each study site were used for *kdr* and *ace.1 ^R^* screening. Table 2 presents the distribution of knock-down (L1014F, L1014S and N1575Y) and *ace.1 ^R^* (G119S) mutations in *An. gambiae s.s.* and *An. coluzzii*. The L1014F kdr mutation was found at various allelic frequencies in *An. gambiae s.s.* and *An. coluzzii* in the three sites. The 1014F allelic frequency was high in both species ranging from 66% to 100% in *An. coluzzii* and from 80% to 83.96% in *An. gambiae s.s*. The 1014S kdr allele was found for the first time in *Anopheles* mosquitoes from Togo (Kovié and Nangbéto). The allelic frequency of this kdr mutation ranged from 5.17 to 13.46% in both *An. gambiae s.s* and *An. coluzzii*. The kdr N1575Y were also detected in the two species with the allelic frequencies ranging from 10.34 to 45.06% in all the sites investigated. *ace.1 ^R^* mutation was mainly found in *An. gambiae s.s* with allelic frequency ranging from 4.87 to 16.66%.

**Table 2.** Resistant allele frequencies in *Anopheles coluzzii* and *Anopheles gambiae s.s* in study sites.

Localities	Species	N	f (1014F)	N	f (1014S)	N	f (1575Y)	N	f (119S)
Kovié	***An. coluzzii***	79	67.72%±10.31	87	5.17%±4.65	81	45.06%±10.84	63	16.66%±10.76
Nangbéto		27	66.67%±17.78	26	13.46%±7.56	29	10.34%±7.76	28	-
Mango		2	100%	2	-	2	-	2	-
Nangbéto	***An. gambiae s.s***	40	80%±12.4	31	6.45%±4.38	41	17.07%±11.66	41	4.87%±2.67
Mango		53	83.96%±9.88	53	-	52	11.53%±8.6	49	7.14%±6.93

NB: Allelic frequencies (f) are given in means ± Standard deviation (SD)

## Discussion

This study provides an update on current levels of resistance to permethrin and deltamethrin and frequencies of the *kdr* and *ace.1 ^R^* mutation in *An. coluzzii* and *An. gambiae s.s* in rural areas of Togo.

*An. coluzzii* and *An. gambiae s.s*. ** were the only *Anopheles* species observed in this study and lived in sympatry at varying frequencies. *An. gambiae s.s* was the most abundant species at the two cereal cultivation sites of Mango and Nangbéto whereas *An. coluzzii* is far more predominant in the rice field of Kovié, supporting findings from previous research in Togo and Benin ^3, 29^. This heterogeneous composition of *An. gambiae* population observed in these localities could be due to a competitive exclusion between the two subspecies ^30^. This study provides updated information on the insecticide resistance profile of *Anopheles gambiae* and the underlying mechanisms involved in rural areas of Togo.

It also provides baseline information on the susceptibility/resistance status of *An. coluzzii* and *An. gambiae s.s* in this location to permethrin and deltamethrin, the insecticides used to impregnate the bed nets freely distributed by NMCP of Togo. The WHO bioassay results indicated a high prevalence of resistance to pyrethroids/DDT in all study sites. This suggests that similar selection pressures are acting on these populations. Recently Ahadji-Dabla *et al*. ^20^ reported similar observation of resistance in *An. gambiae s.l* populations to DDT, deltamethrin and permethrin in Lomé. The proportion of *An. gambiae* surviving to permethrin exposure has increased slightly in Kovié compared with that in previous studies conducted in the same area: In 2009, Ahadji-Dabla *et al.* ^21^ reported mortality of 56%, while any mortality was recorded in this study. This observation suggests that pyrethroids resistance had significantly increased in this area over the last five years. It probably indicates that the selection pressure is not high but could change with the intensive and uncontrolled used of chemical and fertilizers in rice production and the mass-distributed of LLINs in this area. Moreover, the implantation of uncontrolled insecticides selling markets in the country especially around rice cultivation areas, contributed to the increase in the resistance ^20^. This can therefore explain the high frequencies of *kdr* resistant alleles found in our study areas. The knock-down resistance gene was the main resistance mechanism found in all assessed mosquito populations. The 1575Y allele was found in both *An. coluzzii* and *An. gambiae s.s* in all the study sites. The frequencies of this mutation were similar to those found in the nearby countries like Benin ^18^ and Burkina-Faso ^17^. This allele was highly distributed in *An. coluzzii* in Kovié (0.45) as previously reported in the rice fields in Northern Benin ^18^. However, the prevalence of this mutation has rapidly increased in the West African region suggesting an ongoing strong selection of the L1014F-N1575Y haplotype in this region. The *kdr* (L1014F) mutation was found at higher frequencies in *An. gambiae s.s* than *An. coluzzii* at all sites. These results confirm those of Dabiré *et al*. ^31^ who found that *An. gambiae s.s* showed the highest levels of resistance than *An. coluzzii*.

This kdr mutation could be responsible for the high resistance observed to permethrin and deltamethrin in *An. gambiae* population from Togo. Hence, this is a serious problem for malaria control programs because the currently widespread distributed nets are impregnated by these insecticides. Fortunately, a recent paper published in Benin revealed that insecticide-treated nets provide protection against malaria to children in an area of insecticide resistance in southern Benin ^32^.

To our knowledge, this study is the first reporting the presence of the L1014S *kdr* mutation in wild *An. gambiae s.l* populations from Togo. The L1014S allele was detected in both *An. coluzzii and An. gambiae s.s.* This allele, originating from East Africa, was recently reported in Benin in *An. arabiensis* ^3^ and in *An. coluzzii* and *An. gambiae s.s* in ** Burkina-Faso ^33^. It is possible that *An. gambiae* populations carrying the *kdr* L1014S mutation might have migrated, through active or/and passive ways, from bordering countries (e.g. Benin, Burkina-Faso) due to intense traffic and exchanges between these countries and Togo.

These findings therefore provide strong evidences on the increasing distribution of the *kdr* mutations among *Anopheles* mosquitoes across Africa, and could be used as baseline data for proper monitoring of this allele in West African countries. Further research should be implemented to provide knowledge on the geographical distribution of L1014S *kdr* allele in West Africa, its role in pyrethroids phenotypic resistance, as well as its impact on the efficacy of pyrethroids treated nets.

In the present study, the G119S mutation was identified in all investigated sites at a relatively low frequency. This is in contrast to previous findings that reported a high frequency of *ace-1 ^R^* mutation in *An. gambiae s.l* from Lomé ^34^. This resistant allele was detected in both species with frequencies ranging from 4.8% to 16.66%. The presence of *ace-1 ^R^* mutations in *An. coluzzii* and *An. gambiae s.s.* has already been reported by Weill *et al.* ^35^ and Djogbenou *et al.* ^36^. The incidence of the G119S mutation in the *An. gambiae s.l* population from Togo suggests a probable resistance to carbamates and organophosphates insecticides. However, this assertion needs to be proved by WHO toxicological tests ^28^. We cannot therefore exclude the possibility that besides the four mutations targeted in these study sites, other enzymes and genetic mechanisms could be contributed to the resistance, as suggested by previous studies in Benin ^18, 37^.

## Conclusion

The present study revealed the widespread of *kdr* 1014F, 1014S and 1575Y as well as the G119 alleles in Togo. For the first time, this gives the evidence of the presence of 1014S *kdr* allele in wild populations of *An. gambiae s.l* from Togo where entomological surveys are scanty. Hence confirming the expansion of pyrethroids resistance alleles in Africa. There is therefore a need for regular updating on the current entomological data for appropriate decision making and proper intervention strategies for malaria vector control in this country.

## Ethics statement and consent

No ethical clearance was required for this study according to the International Institute of Tropical Agriculture (IITA) Ethical Committee (IITA, 08 P.O. Box 0932, Tri-Postal, Cotonou, Benin). However, consent of the community leaders was sought prior to mosquito larva and adult collections in the community. We explained our study to the communities and household heads. Verbal and written consents of household heads were therefore obtained prior for mosquito collection

## Data availability

All data generated and analyzed during this study is included in the published article. Raw data are available from Open Science Framework: Dataset 1. First report of the presence of L1014S Knockdown-resistance mutation in *Anopheles gambiae s.s* and *Anopheles coluzzii* from Togo, West Africa, http://doi.org/10.17605/OSF.IO/M3G4P ^38^
