# Supplementary material for: First report of the presence of L1014S Knockdown-resistance mutation in Anopheles gambiae s.s and Anopheles coluzzii from Togo, West Africa
Source: Wellcome Open Res. 2018 Mar 19;3:30. [Version 1] doi: 10.12688/wellcomeopenres.13888.1 (PMC5909049; doi:10.12688/wellcomeopenres.13888.1)
Supplement: Peer review history

# Peer review: Referee response for version 1

**DOI:** 10.21956/wellcomeopenres.15098.r32170

**Published:** 2018-04-18

**Authors:** Luc Djogbenou, Aki Victorien Constant Edi

**Affiliations:** 1 Institut Régional de Santé Publique/Université d'Abomey-Calavi (IRSP/UAC), Ouidah, Benin; 2 Research and Development Department, Centre Suisse de Recherches Scientifiques en Côte d’Ivoire, Abidjan, Cote d'Ivoire

**Introduction**

 1- the sentence « However, malaria vectors were found resistant to all insecticide

 classes used in public health interventions in West Africa » need to be referenced.

 2- Add a sentence in the introduction describing the G119S mutation causing resistance to organophosphates and carbamates as done for others target side modification mechanisms.

**Materiel & Methods**

 The attempt was made by the authors to explain the methods used and analysis performed.

**Results**

 The authors stated in their Statistical analysis that « According to Hardy-Weinberg equilibrium using the Had2know online statistical software, calculated genotype frequencies of L1014F, L1014S, N1575Y and G119S were confirmed and compared between *An. coluzzii *and *An. gambiae s.s. *with Chi-square test. ».

 By analysing the table bearing the frequencies data, these statistical analysis results were not clear. Furthermore, it not easy to understand how the authors can inferred the standard deviation (SD) from the calculations of resistant allele frequencies.

 All these remarks need to be clarified by the authors.

**Discussion**

 The sentence need to be rephrased and be more specific « This study provides an update on current levels of resistance to permethrin and deltamethrin and frequencies of the

*kdr *and *ace.1R *mutation in *An. coluzzii *and *An. gambiae s.s *in rural areas of Togo » It better to precise the location where the study was performed.

 The sentence « The WHO bioassay results indicated a high prevalence of resistance

 to pyrethroids/DDT in all study sites » need to be rephrased using « high level of resistance » instead of « high prevalence of resistance »

 The authors stated: « The WHO bioassay results indicated a high prevalence of resistance to pyrethroids/DDT in all study sites. This suggests that similar selection pressures are acting on these populations ». I have a concern with the logical between these two sentences. In my opinion, the high level of resistance displayed to pyrethroids cannot allow the authors to state « that similar selection pressures are acting on these populations »

 The authors stated:  « In 2009, Ahadji-Dabla *et al.*21 reported mortality of 56%, while any mortality was recorded in this study » this sentence need to be rephrased to « In 2009, Ahadji-Dabla *et al.*21 reported mortality of 56%, while no mortality was recorded in this study 

 The authors stated in their discussion « The proportion of *An. gambiae *surviving to permethrin exposure has increased slightly in Kovié compared with that in previous studies conducted in the same area: In 2009, Ahadji-Dabla *et al.*21 reported mortality of 56%, while any mortality was recorded in this study. This observation suggests that pyrethroids resistance had significantly increased in this area over the last five years. It probably indicates that the selection pressure is not high but could change with the intensive and uncontrolled used of chemical and fertilizers in rice production and the mass-distributed of LLINs in this area. »

 This part of the discussion appears to be confused for me.

 Firstly, when they attempted to compare the mortality observed in this study to that of previous studies, the authors need to pay attention to the number of mosquitoes used for WHO bioassay compared to that which was used in the previous work. For me it seems that these numbers are largely different. They can attempt to speculate but not conform.

 Secondly, it is very hard to understand how they can infer from the sentence "This observation suggests that pyrethroids resistance had significantly increased in this area over the last five years" that "the selection pressure is not high but could change with the intensive and uncontrolled used of chemical ........). I'm confused when referring to the statement of the authors in the previous sentences.

 The title of the manuscript is "First report of the presence of L1014S Knockdown-resistance mutation in *Anopheles gambiae* s.s and *Anopheles coluzzii* from

 Togo, West Africa" By analysing the discussion length, we can observe that only one paragraph was devoted for the discussion related to the presence of L1014S Knockdown-resistance mutation in *Anopheles gambiae* s.s and *Anopheles coluzzii* from Togo.

 I strongly recommend the authors to rewrite the discussion and focus their discussion on idea related to the title or they can change the title of the manuscript according the discussion content.

We have read this submission. We believe that we have an appropriate level of expertise to confirm that it is of an acceptable scientific standard, however we have significant reservations, as outlined above.

# Peer review: Referee response for version 1

**DOI:** 10.21956/wellcomeopenres.15098.r32167

**Published:** 2018-04-17

**Authors:** Nafomon Sogoba

**Affiliations:** 1 International Center of Excellence in Research (ICER-Mali), Faculty of Medicine and Odontostomatology (FMOS), University of Sciences, Techniques and Technologies of Bamako, Bamako, Mali

The manuscript “First report of the presence of L1014S Knockdown-resistance mutation in *Anopheles gambiae s.s* and *Anopheles coluzzii* from Togo, West Africa” has updated data on pyrethroids/DDT resistance in the *An. gambiae s.l* population from Togo. In particular, it investigated the distribution of the kdr L1014F, L1014S and N1575Y alleles and ace.1R mutations in different ecological settings of Togo.

 General comments

 Overall the manuscript was very well written and I have only few comments:

1. Is it appropriate to compare results from mosquitoes collected in 2013 to those collected 2 years later (2015) when we know that insecticide resistance is evolving quickly?
2. How can you explain the high difference between the mortality rates of Deltamethrin and Permethrin in Nangbéto?

I have read this submission. I believe that I have an appropriate level of expertise to confirm that it is of an acceptable scientific standard.

# Peer review: Referee response for version 1

**DOI:** 10.21956/wellcomeopenres.15098.r32168

**Published:** 2018-04-03

**Authors:** Christophe Antonio-N'kondjio

**Affiliations:** 1 Malaria Research Laboratory, Organisation for Coordination in the Fight Against Endemic Diseases in Central Africa, Yaoundé, Cameroon

**General observations**

 The study by Djègbé et al provides information on mosquito susceptibility and insecticides resistance in Togo and is important for the improvement of vector control strategies on the field. There is however a need to improve the english level.

**Abstract**

 Background: “ This paper reported an updated data on pyrethroids/DDT resistance in the population An. gambiae s.l from Togo.” Not clear please rephrase the sentence

 Methods: “Resistance to DDT, permethrin and deltamethrin was screened in F1 progeny of collected mosquitoes using WHO susceptibility tests.” Change to “susceptibility to DDT, permethrin, and deltamethrin was screened in the F1 progeny of collected mosquitoes using WHO susceptibility bioassay tests”.  

 Results: “The Kdr N1575Y was present at various…” change to “ The N1575Y mutation frequency varied from 10% to 45%”.

 Conclusion: In conclusion give the importance of your findings for vector control

**Introduction**

 “Malaria remains the fourth leading cause mortality ….” Change to “Malaria remains the fourth leading cause of mortality ….”

 “However, malaria vectors were found resistant to all insecticide classes used in public health interventions in West Africa.” Please add a reference at the end of this sentence.

 The authors also need to provide information on the situation of malaria (morbidity, mortality) and insecticides resistance in Togo. What is the level of insecticide resistance in Togo, what it is the level of coverage of the population by treated nets (this information could be extracted from the annual world malaria report)

**Method**

 Mosquito collection and rearing

 The authors say mosquito collections were undertaken from December 2013 to April 2015. This sentence is misleading they authors have to say as presented in table 1 that, mosquitoes were collected in Kovié in December 2013 and in Nangbéto and Mango in April 2015.

 Collections were conducted during how many days/site/sampling period? Please add the information

 The progeny of how many female were used for insecticide susceptibility tests because the result could be biased if just a few female progeny were used

 Figure 1 please add the position of Togo in Africa

**Results**

 Can the number of mosquito collected using electric aspirators during each period in each site be added the number who successfully laid eggs and the total number of mosquito who emerged from the females.

 The author say in the statistical analysis section “According to Hardy-Weinberg equilibrium using the Had2know online statistical software, calculated genotype frequencies of L1014F, L1014S, N1575Y and G119S were confirmed and compared between An. coluzzii and An. gambiae s.s. with Chi-square test.” In the result section, no data of the comparison of genotypes between An. gambiae and An. coluzzii is provided please add these data.

**Discussion**

 Mosquito used for bioassay were the progeny of females collected resting indoor it is possible that mosquitoes resting indoor may be more tolerant to insecticide than the general population. Following this it is possible that increased level of resistance recorded could be due to the use of this fraction of the population this need to be clearly stated in the discussion.

 Also the fact that no *An. arabiensis* was recorded during the study could be due to the fact that the sampling concerned mosquitoes resting indoor whereas this mosquito is known to be highly exophilic the absence of *An. arabiensis* could result from a sampling bias this has to be included

**Source of data underlying the results available**

 The authors need to include information on the genotypes to the kdr gene in the different sites

I have read this submission. I believe that I have an appropriate level of expertise to confirm that it is of an acceptable scientific standard.
